# Systematic review and meta-analysis of the sero-epidemiological association between Epstein-Barr virus and rheumatoid arthritis

**DOI:** 10.1186/s13075-015-0755-6

**Published:** 2015-09-29

**Authors:** Robert J. Ball, Alison Avenell, Lorna Aucott, Peter Hanlon, Mark A. Vickers

**Affiliations:** Health Services Research Unit, Division of Applied Health Sciences, University of Aberdeen, Foresterhill, AB25 2ZD Aberdeen, UK; Research Health Services Research Unit, Division of Applied Health Sciences, University of Aberdeen, Foresterhill, Aberdeen, AB25 2ZD UK; School of Medicine and Dentistry, University of Aberdeen, Foresterhill, Aberdeen, AB25 2ZD UK; Division of Applied Medicine, University of Aberdeen, Foresterhill, Aberdeen, AB25 2ZD UK; Blood Transfusion Centre, Foresterhill Road, Aberdeen, AB25 2ZW UK

## Abstract

**Introduction:**

Infection with Epstein-Barr virus (EBV) has been suggested to contribute to the pathogenesis of autoimmune diseases, including rheumatoid arthritis (RA). We sought to determine whether prior infection with the virus occurs more frequently in patients with RA compared to controls.

**Methods:**

We performed a systematic review and meta-analyses of studies that reported the prevalence of anti-EBV antibodies in the sera of cases with RA and controls by searching Medline and Embase databases from 1946 to 2014, with no language restriction. Mantel-Haenszel odds ratios for the detection of anti-EBV antibodies were calculated, and meta-analyses conducted. Quality assessments were performed using a modified version of the Newcastle-Ottawa scale.

**Results:**

Twenty-three studies were included. Quality assessment found most studies reported acceptable selection criteria but poor descriptions of how cases and controls were recruited. When all studies were included, there was a statistically significant higher seroprevalence of anti-VCA IgG in patients with RA compared to controls with an odds ratio (OR) of 1.61 (95 % confidence interval (CI) 1.05–2.46, *p* = 0.03), which is a similar-sized summary OR to that reported for systemic lupus erythematosus (SLE). However, when studies were restricted to those reporting more plausible levels of exposure to EBV in the control groups, no significant association was apparent, OR 1.47 (95 % CI 0.88–2.46, *p* = 0.14). Using anti-EBNA 1 or anti-EA IgG as markers of previous infection also did not yield significant associations (OR 1.05, 95 % CI 0.68–1.61, *p* = 0.82; OR 2.2, 95 % CI 0.86–5.65, *p* = 0.10 respectively).

**Conclusions:**

Overall, these findings do not demonstrate an association between EBV seroprevalence and RA and therefore do not support the hypothesis that prior infection with EBV predisposes to the development of RA. This contrasts with meta-analyses that indicate EBV infection is associated with multiple sclerosis and SLE.

**Electronic supplementary material:**

The online version of this article (doi:10.1186/s13075-015-0755-6) contains supplementary material, which is available to authorized users.

## Introduction

The causes of rheumatoid arthritis (RA) remain uncertain. Several genetic loci involved in immune responses have been identified [1], but environmental determinants have been harder to identify. An increased risk in cigarette smokers [2] represents the only generally accepted such factor [3]. Claims that prior infection with Epstein-Barr virus (EBV) is important are of particular interest, as an association has been repeatedly demonstrated for multiple sclerosis (MS) [4] and a recent meta-analysis implicated the virus in the pathogenesis of systemic lupus erythematosus (SLE) [5].

Control of EBV infection has been suggested to be impaired in RA patients [6] with studies using real-time polymerase chain reaction (rt-PCR) techniques demonstrating EBV DNA loads in peripheral blood mononuclear cells greater than ten times those of normal controls [7]. EBV has been hypothesised to cause RA through several mechanisms. Most notable is molecular mimicry, which was first suggested following the identification of serum from RA patients exhibiting reactivity against a nuclear antigen in EBV-infected lymphocytes, called the RA nuclear antigen (RANA) [8]. A second example of molecular mimicry was shown between the QKRAA amino acid motif of the β-chain of HLA-DR4 and EBV glycoprotein 110 (gp110). EBV infection in normal individuals triggers production of antibodies to gp110, which were demonstrated in vitro to bind to HLA-DR4 [9]. Additional cross-reactivity has been shown between the EBV peptide p107 and anti-denatured collagen and anti-keratin antibodies [10]. Synovial T cells from RA patients have been shown to recognise and be activated by peptides from the EBV transactivators (MZLF1 and BMFL1) [11]. In addition to molecular mimicry, suggested mechanisms for EBV causing immune-mediated disease [12] include mistaken self [13], bystander damage surrounding EBV reactivation [14], immortalisation of B cells secreting self-reactive antibodies [15] and the resetting of the immune system to favour more active global immunity against, with correspondingly less tolerance to, antigens [16].

Laboratory identification of EBV infection typically relies on detecting antibodies to EBV antigens, which include: EBV nuclear antigen (EBNA) -1, viral capsid antigen (VCA), early antigen complex-diffuse (EA-D) and early antigen complex-restricted (EA-R). Antibodies to these EBV antigens can be utilised to identify the stage of EBV infection. Antibodies to EA are thought to indicate active EBV replication, whereas antibodies to EBNA-1 and VCA persist for the life of the host.

Several researchers have reported higher proportions of patients with RA having antibodies to EBV than controls. However, these studies have been limited by small sample sizes and have been contradicted by other studies reporting no association. To date the authors are aware of no systematic reviews examining the association between RA and previous infection with EBV. We and others have previously reported systematic reviews demonstrating significant associations between the presence of antibodies to EBV and both MS [4] and SLE [5]. However, it is uncertain whether this association applies to other immune-mediated diseases. In this study we present the first systematic review and meta-analysis of seropositivity for EBV among RA patients compared to controls.

## Methods

### Search strategy

The Ovid search tool was used to explore Medline and EMBASE databases with a structured use of MeSH and Emtree headings for RA and EBV (Additional file 1). A search was performed from 1946 to week 1 December 2014 in Medline and from 1984 to week 1 December 2014 in EMBASE. No restrictions were made to language or country of study origin. Cohort or case–control studies were included in this systematic review, which recruited patients and controls of any age with RA and reported antibodies to any of the following EBV antigens: VCA (viral capsid antibody), EA (early antigen), EBNA (Epstein-Barr virus nuclear antigen) -1 or -2. Non-human studies were excluded, as were those reporting only immunoglobulin M (IgM) antibodies.

**Fig. 1:**
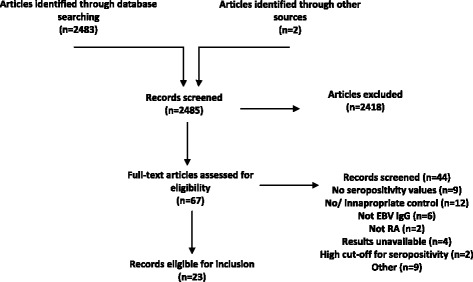
Flow chart. Following removal of duplicates, the search produced 2482 abstracts and two articles were identified from references of review articles. Sixty-seven were considered for inclusion and full texts accessed. Forty-four articles were excluded due to a number of the following reasons: no seropositivity values (n = 9); no or inappropriate controls (n = 12); no EBV IgG or IgA data (n = 6); and non-RA data (n = 2). Full results were not available for four articles and authors were contacted for available data, however, there were insufficient data provided in each case and all were excluded. The remaining 23 studies were considered eligible for inclusion and are summarised in Additional file 2. *EBV* Epstein-Barr virus, *Ig* immunoglobulin, *RA* rheumatoid arthritis

One reviewer read the abstracts and selected those for full text examination (Fig. 1). Full texts of these articles were read, with those meeting the inclusion criteria considered for data extraction. Authors were contacted when data were not fully available. A selection of 200 abstracts from the Medline search were reviewed by a second reviewer to examine for inter-reviewer variation, with those for which full texts that should be sought and those likely to be eligible for inclusion highlighted.

### Quality assessment

Included studies were evaluated using the Newcastle-Ottawa assessment scale [17], which was adapted to offer one star for conducting the analysis in a clinical laboratory (away from investigators), one star for mentioning explicit laboratory cutoffs for seropositivity and one star for reporting whether or not there were missing data.

### Data analysis

Two of the authors independently extracted data from each included study and any differences were discussed, and resolved by discussion. One native speaker extracted data from the Turkish [18] and Chinese [19] articles. The odds ratios (ORs) of seropositivity to EBV were calculated for each of the anti-EBV antibodies using Review Manager (RevMan version 5.2. Copenhagen: The Nordic Cochrane Centre, The Cochrane Collaboration, 2012). Odds ratios for each antibody were combined in a meta-analysis. As we anticipated considerable study heterogeneity, we utilised a conservative random-effects model with a 95 % confidence interval (CI). I^2^ was used to assess heterogeneity between studies [20].

Post hoc subgroup analysis testing was performed to compare the OR of VCA seropositivity in the following categories to investigate potential sources of heterogeneity: studies with greater than 80 % VCA seropositivity in the control group; studies with both age- and sex-matched controls and studies with community controls.

## Results

Following removal of duplicates, the search produced 2482 abstracts, from which 67 were considered for inclusion and full texts accessed. A kappa statistic of agreement (calculated using IBM SPSS Statistics for Windows, Version 22 of SPSS: IBM Corp, Armonk, NY, USA) was high at 87 % (*p* = <0.001). One author read each of the 67 articles, with 40 meeting inclusion criteria, and these were read by one of two additional authors for consideration. One article was written in Chinese and one article in Turkish with a single researcher reviewing each of these articles. Four articles written in Russian were reviewed by another researcher with none meeting the inclusion criteria due to a lack of appropriate controls (n = 2), no IgG-specific data on EBV (n = 1) and data for leucocytes only (n = 1). Two papers (Thomas et al. [21] and Alspaugh et al. [22]) reported EBV VCA IgG as seropositive at titres <1/320 leading to low control group seropositivity and so the authors excluded these two studies. In total seventeen articles were excluded with agreement between authors, leaving 23 articles for the meta-analysis (Fig. 1).

The 23 articles are summarised in Additional file 2 [6, 18, 19, 23–42]. All were case–control studies; no cohort studies were identified. The median sample size for the disease group was 50 (range 20–140) and 43 for the control group (range 14–245). Quality assessment is provided in Table 1. Three studies provided no diagnostic criteria for the diagnosis of rheumatoid arthritis, while the remaining 20 utilised a form of either the American College of Rheumatology (ACR) or American Rheumatology Association criteria. The reported matching of controls was poor, with only seven studies controlling for both age and sex, and two controlling for age only. One study by Lunemann et al*.* [24] reported sex matching: however, the presented demographics exhibited large disparities between control and RA groups and so it was considered as not sex-matched. The reporting of blinding of researchers was also poor, with only one study blinding analysts to patient group. Reporting of data was often unsatisfactory, with only nine detailing the cutoff values for seropositivity (Table 1).

**Table 1:** Adapted Newcastle-Ottawa quality assessment

Study ID	S1	S2	S3	S4	C1	C2	E1a	E1b	E2	E3
Ferrell and colleagues^23^	*	-	-	-	-	-	-	-	*	-
Lunemann and colleagues^24^	*	-	*	-	*	-	-	-	*	-
Saal and colleagues^25^	*	-	-	-	*	-	-	*	-	-
Yazbek and colleagues^26^	*	*	-	*	*	*	-	-	*	-
Mouzavi-Jazi and colleagues^27^	-	-	-	*	-	-	-	-	*	-
Blaschke and colleagues^28^	*	-	*	*	*	*	-	-	*	-
Venables and colleagues 1981^29^	*	-	-	-	-	-	-	*	*	-
Venables and colleagues 1985^30^	*	-	-	*	-	-	-	-	*	-
Sculley and colleagues^31^	*	-	*	-	-	-	-	-	*	-
Shirodaria and colleagues^32^	*	-	-	*	*	*	-	*	*	-
Silverman and colleagues^33^	*	-	-	*	*	*	-	-	*	-
Ng and colleagues^34^	*	-	-	-	-	-	-	-	-	-
Musiani and colleagues^35^	*	-	-	*	*	*	-	*	*	-
Yao and colleagues^36^	*	-	-	-	*	-	-	-	*	-
Philips and colleagues^37^	*	-	*	*	-	-	-	-	*	-
Nakabayashi and colleagues^38^	*	-	-	-	*	*	-	*	-	-
Us and colleagues^18^	*	-	*	*	*	-	-	-	*	*
Youinou and colleagues^39^	*	-	*	-	-	-	-	*	*	-
Draborg and colleagues^40^	*	-	-	-	-	-	-	*	*	-
Jorgensen and colleagues^41^	*	*	*	*	*	*	-	*	*	-
Zhang and colleagues 1999^19^	*	-	*	*	-	-	*	*	*	-
Zhang and colleagues 1993^6^	*	-	*	*	-	-	-	-	*	-
Davies and colleagues^42^	*	-	-	-	*	-	-	-	*	-

Adapted Newcastle-Ottawa assessment scale. *S1* objective case definition used, *S2* cases consecutively recruited or obviously representative, *S3* community controls, *S4* controls specified as having no history of disease, *C1* age-matched control, *C2* controls matched for additional factor, *E1a* conducting analysis in a clinical laboratory (away from investigators), *E1b* explicit laboratory cutoffs for seropositivity mentioned, *E2* same method of ascertainment for cases and controls, *E3* missing data reported

### Anti-viral capsid antigen IgG

Eighteen studies reported data on the presence of anti-VCA IgG, including a total of 943 RA cases and 891 controls, of which eight demonstrated higher rates of seropositivity in RA patients compared to controls. The summary OR demonstrated a statistically significant increased seropositivity in RA patients compared to controls with OR 1.61 (95 % CI 1.05–2.46, *p* = 0.03) and *I*^2^ = 1 % (*p* = 0.44) (Figure S1 in Additional file 1).

**Fig. 2:**
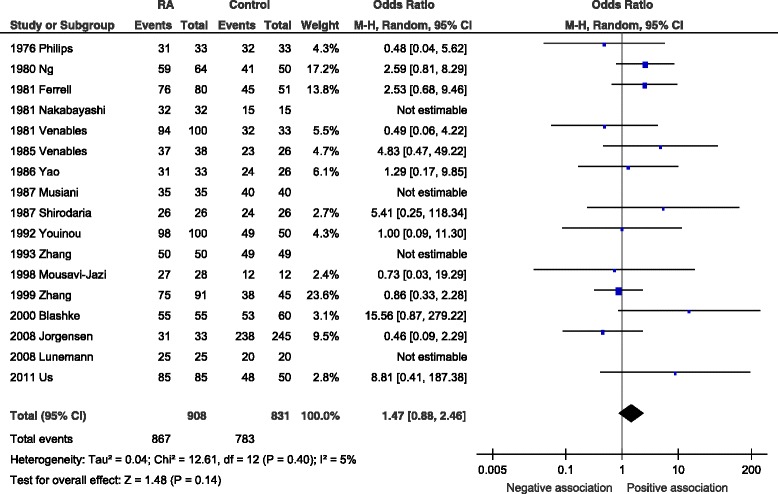
Forest plot for anti-VCA IgG excluding Saal et al*.* Random-effects meta-analysis of seroprevalence of anti-viral capsid antigen (VCA) immunoglobulin G (IgG) between rheumatoid arthritis casesand controls excluding Saal et al. [25]. *CI* confidence interval, *M-H* Mantel-Haenzsel, *RA* rheumatoid arthritis

However, on inspection of the data it was apparent that one study (Saal et al*.* [25]) reported substantially lower rates of antibody detection in both cases and controls than the other studies and indeed, all the studies included in previous studies of MS and SLE. On rereading this article, no apparent cause was identified. We consider these rates to be implausibly low and suspect that there were problems in performance of the laboratory assays. Following exclusion of this article there was no significant difference between RA patients and control group EBV VCA IgG seroprevalences with OR 1.47 (95 % CI 0.88–2.46, *p* = 0.14) and I^2^ = 5 % (*p* = 0.40) (Fig. 2).

Including only studies with both age- and sex-matched controls led to a significant difference for VCA IgG seroprevalence between RA patients and controls with OR 9.27 (95 % CI 1.64– 52.6, *p* = 0.01). However this analysis was based on only six studies, although there was a low degree of heterogeneity demonstrated (*I*^*2*^ = 0 %, *p* = 0.88) (Figure S2 in Additional file 1). Restricting the analysis to studies with community controls did not to lead to a statistically significant difference with OR 2.02 (95 % CI 0.64–6.41, *p* = 0.23).

### Anti-Epstein-Barr virus nuclear antigen-1 IgG

**Fig. 3:**
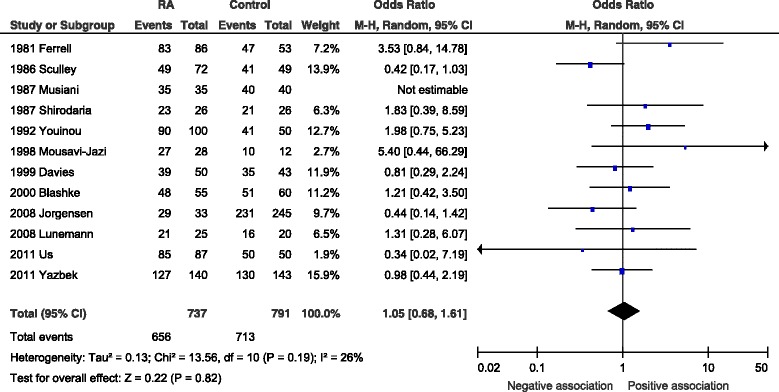
Forest plot for anti-EBNA-1 IgG. Random-effects meta-analysis of seroprevalence of anti-Epstein-Barr virus nuclear antigen-1 (EBNA-1) immunoglobulin G (IgG) between rheumatoid arthritis cases and controls. *CI* confidence interval, *M-H* Mantel-Haenzsel, *RA* rheumatoid arthritis

Twelve studies reported anti-EBNA-1 serology, with a total of 737 RA patients and 791 controls. Six studies demonstrated higher seropositivity in RA patients. However, there was no statistically significant difference between cases of RA and controls with OR 1.05 (95 % CI 0.68–1.61, *p* = 0.82). The samples showed a low level of heterogeneity *I*^2^ = 26 % (*p* = 0.19) (Fig. 3).

### Anti-early antigen IgG

**Fig. 4:**
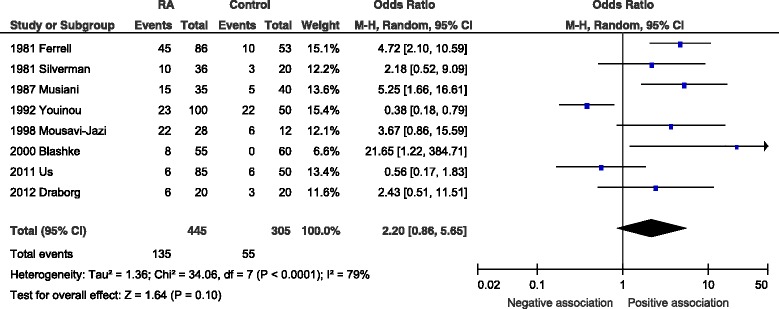
Forest plot for anti-EA IgG. Random-effects meta-analysis of seroprevalence of anti-early antigen (EA) immunoglobulin G (IgG) between rheumatoid arthritis cases and. *CI* confidence interval, *M-H* Mantel-Haenzsel, *RA* rheumatoid arthritis

Eight studies reported anti-early antigen (anti-EA) serology, with 445 RA cases and 305 controls. A higher degree of seropositivity was demonstrated in the RA group compared to the controls. However this did not reach statistical significance with an OR of 2.2 (95 % CI 0.86–5.65, *p* = 0.10) and the studies showed high heterogeneity with *I*^2^ = 79 % (*p* = 0.0001) (Fig. 4).

## Discussion

This meta-analysis of case–control studies investigating the association between RA and serological markers of EBV infection failed to show a significant association between the disease and anti-VCA IgG (OR 1.47, 95 % CI 0.88–2.46, *p* = 0.14), anti-EBNA1 IgG (OR 1.05, 95 % CI 0.68–1.61, *p* = 0.82) or anti-EA (OR 2.2, 95 % CI 0.86–5.65, *p* = 0.10). This analysis is, to our knowledge, the first attempt to combine such estimates of association with RA in a meta-analysis and should therefore provide a more robust estimate of the association than individual studies, which have tended to include relatively small numbers of participants.

Strengths of this study include its comprehensive search strategy, without language restriction. Conversely, meta-analyses such as these are susceptible to publication bias, but there is no suggestion of this on a funnel plot of those studies included in the anti-VCA. Of further concern is the high level of heterogeneity between the ORs of the individual studies, the lack of consistency in matching of cases and controls, and the paucity of reporting of recruitment and laboratory methodology, as seen in the quality assessment. We attempted to control for these factors by analysing only studies with community controls; however, this limited the overall RA population studied for VCA and EBNA-1 antibodies to seven studies, with the results showing no difference between seropositivity for these two groups. Thus further examination of this topic with well-constructed, larger case–control studies would be valuable in determining whether EBV infection helps predispose to RA. A further factor that might explain why antibodies to EBV are detected more frequently in patients with RA compared to controls is that RA is an inflammatory condition, with high concentrations of immunoglobulins, including those directed against EBV (Thomas et al*.* [21] and Alspaugh et al*.* [22]). Thus failures to detect low titre antibodies would be expected to occur more commonly in the control groups, although the importance of this effect is difficult to quantify. A further observation supporting our conclusion of no association between RA and EBV is that the two conditions do not occur together more often than that expected by chance [43].

We hoped to find prospective studies that would allow analysis of the relative timing of infection and the development of RA, but our search revealed none. Nevertheless, infection in the general population occurs generally below the age of 20 [43] and so before the onset of symptoms of RA.

The results from this meta-analysis contrast with those examining the role of EBV exposure in MS and SLE. For MS, several meta-analyses have confirmed a robust association, with summary odds ratios of approximately 4–5 [4]. Indeed, it has been suggested that the few seronegative individuals arise because of low sensitivities of the assays used to detect anti-EBV antibodies [44], with enzyme-linked immunosorbent assays (ELISAs) performing worse than immunofluorescent techniques. Examination of our data shows no systematic difference in the odds ratios reported by studies reporting the use of these techniques (Additional file 2).

It is widely accepted that it is difficult to develop MS unless infected by EBV. For SLE, we also reported statistically significant odds ratios for the detection of antibodies to VCA (2.0) and EA in SLE, although that for anti-EBNA1 did not reach significance [5]. Overall, it seems likely that infection by EBV does predispose to the development of SLE, but this conclusion should not be regarded as proven.

If it is accepted that EBV plays an important role in the pathogenesis of MS, probably SLE, but not RA, what are the implications for the mechanisms that underlie these associations? The most popular mechanism invoked to explain autoimmune disease after infections is molecular mimicry, which is usually conceived as a specific pathogen-immune disease pairings. Our findings with RA are therefore consistent with this mechanism. On the other hand, both the hypothesis that EBV immortalises self-reactive B cells that would otherwise be deleted [15] and the idea that EBV resets the global immune system to a more active state [16] imply that EBV infection should cause a generic predisposition to autoimmune disease, which is difficult to reconcile with the findings of the present study. Two further mechanisms suggested to explain the association are mistaken self [36] and bystander damage surrounding EBV reactivation [14], both of which are consistent with the disease associations being confined to specific diseases.

It should be highlighted that a lack of an association between antibodies to EBV and RA does not preclude a role for EBV in the disease as there are other lines of evidence for this [45]. For instance, if the effect were limited to only subtypes of disease or carriers of specific genetic loci, for instance only particular HLA types, then a causal association would not be identified by the numbers included in this meta-analysis.

## Conclusions

In summary, we performed a systematic review and meta-analysis of studies reporting antibodies to EBV antigens in patients with rheumatoid arthritis compared to controls. There was no overall statistically significant association between the presence of antibodies and the disease, but most of the studies had limitations and further data are needed.

## Additional files

Additional file 1: Table S1. Search strategy for systematic review; **Figure S1.** Forest plot anti-VCA IgG including excluded article by Saal et al. [25]; **Figure S2.** Forest plot anti-VCA IgG for age- and sex-matched studies; **Figure S3.** Forest plot anti-VCA IgG for community-controlled studies; study protocol; data extraction form; adapted Newcastle-Ottawa quality assessment scale. (PDF 440 kb) Additional file 2: Table S2. Characteristics of 22 included studies. ^a^Demographics of 57 RA patients and 16 other arthritides not stated, total population provided. ^b^Details not reported. ^c^ELISA (EA, VCA), immunofluorescence (EBNA-1). ^d^Immunoblotting used for EBNA-2a, 2b and -1, immunofluorescence used for VCA and EA. (XLSX 13 kb)

## Abbreviations

CI: confidence interval
EA: early antigen
EBNA: Epstein-Barr virus nuclear antigen
EBV: Epstein-Barr virus
ELISA: enzyme-linked immunosorbent assay
Ig: immunoglobulin
MS: multiple sclerosis
OR: odds ratio
RA: rheumatoid arthritis
RANA: rheumatoid arthritis nuclear antigen
rt-PCR: real-time polymerase chain reaction
SLE: systemic lupus erythematosus
VCA: viral capsid antibody
