# Supplementary material for: Systematic review and meta-analysis of the sero-epidemiological association between Epstein-Barr virus and rheumatoid arthritis
Source: Arthritis Res Ther. 2015 Sep 29;17:274. doi: 10.1186/s13075-015-0755-6 (PMC4587583; doi:10.1186/s13075-015-0755-6)
Supplement: Additional file 1: Table S1. — Search strategy for systematic review; Figure S1. Forest plot anti-VCA IgG including excluded article by Saal et al. [25]; Figure S2. Forest plot anti-VCA IgG for age- and sex-matched studies; Figure S3. Forest plot anti-VCA IgG for community-controlled studies; study protocol; data extraction form; adapted Newcastle-Ottawa quality assessment scale. (PDF 440 kb) [file 13075_2015_755_MOESM1_ESM.pdf]

Database	Rheumatoid arthritis	Epstein-barr virus
Medline MeSH headings	1. exp Arthritis, Rheumatoid/	1. exp epstein-barr virus infections/ or infectious mononucleosis/<br>2. exp Herpesvirus 4, Human
Embase Emtree headings	1. exp rheumatoid arthritis<br>2. rheumatoid nodule.tw.<br>3. exp sjoegren syndrome<br>4. still$ disease.tw.<br>5. pneumoconiosis	1.epstein barr virus<br>2. exp epstein-barr virus infection<br>3. herpes virus 4.tw.
Text words (used both databases)	1. (sicca adj2 syndrome).tw.<br>2. (caplan$ adj2 syndrome).tw.<br>3. (rheumat$ adj2 arthrit$).tw.<br>4. (sjogren$ adj2 syndrome).tw.<br>5. (felty adj2 syndrome).tw.	1. herpesvirus 4.tw.<br>2. mononucleosis.tw.<br>3. kissing disease.tw.<br>4. paul bunnell.tw.<br>5. monospot.mp.<br>6. glandular fever.tw.<br>7. epstein-barr.tw.<br>8. ebv.tw.

Supplemental Table 1 - Search strategy for systematic review

	RA		Control			Odds Ratio
**Study or Subgroup**	**Events**	**Total**	**Events**	**Total**	**Weight**	**M-H, Random, 95% CI**
1976 Philips	31	33	32	33	3.0%	0.48 [0.04, 5.62]
1980 Ng	59	64	41	50	13.3%	2.59 [0.81, 8.29]
1981 Ferrell	76	80	45	51	10.4%	2.53 [0.68, 9.46]
1981 Nakabayashi	32	32	15	15		Not estimable
1981 Venables	94	100	32	33	3.9%	0.49 [0.06, 4.22]
1985 Venables	37	38	23	26	3.4%	4.83 [0.47, 49.22]
1986 Yao	31	33	24	26	4.4%	1.29 [0.17, 9.85]
1987 Musiani	35	35	40	40		Not estimable
1987 Shirodaria	26	26	24	26	1.9%	5.41 [0.25, 118.34]
1992 Youinou	98	100	49	50	3.1%	1.00 [0.09, 11.30]
1993 Zhang	50	50	49	49		Not estimable
1998 Mousavi-Jazi	27	28	12	12	1.7%	0.73 [0.03, 19.29]
1999 Saal	20	35	23	60	24.6%	2.14 [0.92, 5.01]
1999 Zhang	75	91	38	45	19.0%	0.86 [0.33, 2.28]
2000 Blashke	55	55	53	60	2.2%	15.56 [0.87, 279.22]
2008 Jorgensen	31	33	238	245	7.0%	0.46 [0.09, 2.29]
2008 Lunemann	25	25	20	20		Not estimable
2011 Tercan	85	85	48	50	2.0%	8.81 [0.41, 187.38]
**Total (95% CI)**		**943**		**891**	**100.0%**	**1.61 [1.05, 2.46]**
Total events	887		806			

Heterogeneity: Tau² = 0.01; Chi² = 13.12, df = 13 (P = 0.44); I² = 1%

Test for overall effect: Z = 2.17 (P = 0.03)

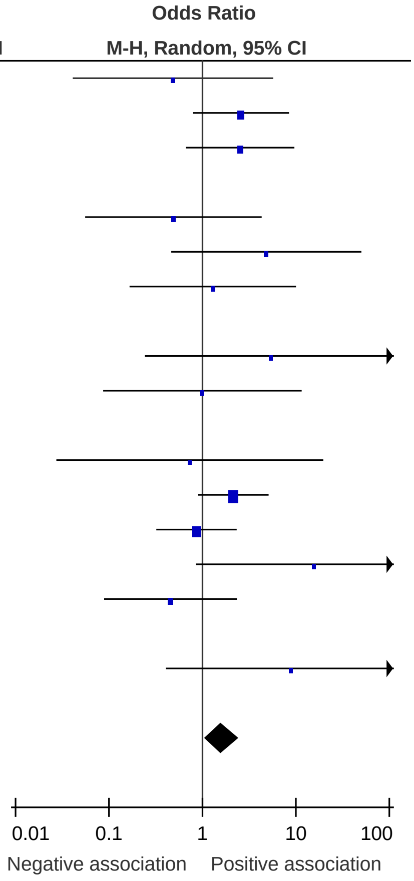

Supplemental Figure 1 - Random effects meta-analysis of seroprevalence of anti-viral capsid antigen IgG between Rheumatoid arthritis cases and controls including excluded paper by Saal *et al*[25]. CI, confidence interval; M-H, Mantel–Haenzsel; RA, Rheumatoid arthritis

	RA		Control			Odds Ratio
Study or Subgroup	Events	Total	Events	Total	Weight	M-H, Random, 95% CI
1981 Nakabayashi	32	32	15	15		Not estimable
1987 Musiani	35	35	40	40		Not estimable
1987 Shirodaria	26	26	24	26	31.6%	5.41 [0.25, 118.34]
2000 Blashke	55	55	53	60	36.1%	15.56 [0.87, 279.22]
2008 Lunemann	25	25	20	20		Not estimable
2011 Tercan	85	85	48	50	32.2%	8.81 [0.41, 187.38]
**Total (95% CI)**		**258**		**211**	**100.0%**	**9.27 [1.64, 52.60]**
Total events	258		200			

Heterogeneity: $Tau^2 = 0.00$; $Chi^2 = 0.25$, df = 2 (P = 0.88); $I^2 = 0\%$

Test for overall effect: Z = 2.52 (P = 0.01)

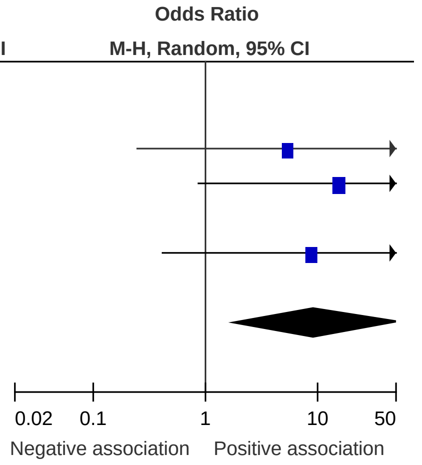

Supplemental Figure 2 - Random effects meta-analysis of seroprevalence of anti-viral capsid antigen IgG between Rheumatoid arthritis cases and controls including studies with both age and sex matched controls. CI, confidence interval; M-H, Mantel–Haenzsel; RA, Rheumatoid arthritis

Study or Subgroup	RA Events	RA Total	Control Events	Control Total	Weight	Odds Ratio M-H, Random, 95% CI
1976 Philips	31	33	32	33	14.8%	0.48 [0.04, 5.62]
1981 Ferrell	76	80	45	51	28.2%	2.53 [0.68, 9.46]
1987 Shirodaria	26	26	24	26	10.6%	5.41 [0.25, 118.34]
1993 Zhang	50	50	49	49		Not estimable
2000 Blashke	55	55	53	60	11.8%	15.56 [0.87, 279.22]
2008 Jorgensen	31	33	238	245	23.8%	0.46 [0.09, 2.29]
2011 Tercan	85	85	48	50	10.8%	8.81 [0.41, 187.38]
**Total (95% CI)**		**362**		**514**	**100.0%**	**2.02 [0.64, 6.41]**
Total events	354		489			

Heterogeneity: $Tau^2 = 0.77$; $Chi^2 = 8.23$, df = 5 (P = 0.14); $I^2 = 39\%$

Test for overall effect: Z = 1.20 (P = 0.23)

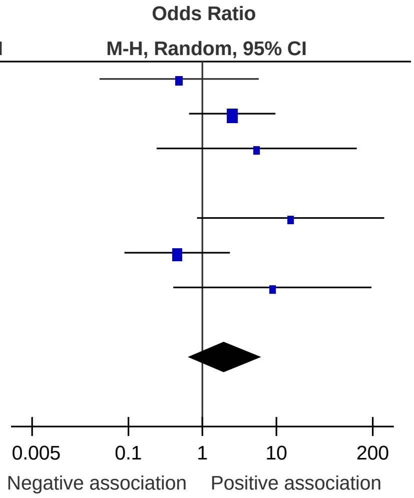

Supplemental Figure 3 - Random effects meta-analysis of seroprevalence of anti-viral capsid antigen IgG between Rheumatoid arthritis cases and controls including studies with community controls. CI, confidence interval; M-H, Mantel–Haenzsel; RA, Rheumatoid arthritis

Study Protocol

Objective

To perform a meta-analysis of case control and cohort studies which measure Epstein-Barr virus (EBV) seropositivity in patients with Rheumatoid Arthritis (RA) compared to healthy controls.

Criteria for consideration of studies Inclusion criteria Studies

- Case control or cohort studies recording EBV seropositivity data
- Studies using any assay for EBV antibodies (VCA, EBNA-1, EA) may be included.
- Studies published in all languages
- Studies from any geographical setting
- Studies from 1966 to present (November 2014)

Participants

- Study subjects of all ages
- Diagnosis according to the American College of Rheumatology (http://www.rheumatology.org/ACR/practice/clinical/classification/ra/ra_2010.asp) or if prior to 1987 then diagnosis according to 1987 Rheumatoid Arthritis Classification.

Exclusion criteria

- Studies which only measure IgM to EBV antigens (detecting recent EBV infection)
- Non-human studies

Search strategy

- A search for studies relevant to rheumatoid arthritis and EBV will be carried out using MEDLINE and EMBASE.
- A combination of specific MeSH terms or EMBASE headings and text words will be used to identify relevant studies.
- Reference lists of relevant articles will be hand searched to identify any further relevant studies

Method of Review

- Non-human studies, studies without a sample population, and case reports will be excluded.
- Abstracts of all possibly relevant articles will be read by two researchers. Discrepancies over which full texts to obtain will be resolved by discussion
- Full texts of all case-control and cohort studies relating RA and EBV will be obtained and the quality of studies assessed by two researchers. Doubts over inclusion will be resolved by discussion.

Data extraction

The following data will be extracted using a standard form

- Year of Study
- Author
- Country (and, where possible, city)
- Sample size
- Age and sex of subjects and controls
- Anti-EBV antibodies assayed (IgG antibodies against VCA, EBNA-1, EA-R)
- Method of serological assay used (Immunofluorescence, ELISA etc.)
- Criteria used to confirm diagnosis of rheumatoid arthritis
- The number of participants seropositive/negative with RA and health controls

Data analysis

The prevalence of EBV positive serology in subjects with autoimmune disease will be compared with that of age matched healthy controls.

- Results with different antibodies (e.g. anti-ENBA 1 IgG and anti-VCA IgG) will be analysed separately
- Age matched studies will be assessed separately
- Studies using community controls will be assessed separately

Statistical analysis

Following data extraction the following statistical analysis will be performed using Review Manager software

- Mantel-Haenzsel odds ratios will be used to compare rheumatoid arthritis patients to health controls with regards to the following: Seropositivity to EBNA-1, seropositivity to VCA, seropositivity to EA-R

Data extraction form

Author Date of extraction:

Year

Study type: Case control/ cohort

EBV serology type: EBNA-1 / EBNA-2 / VCA / EA / other

Cases

Total number	
Male	
Female	
Age range	
Age median	
Age SD	

Control

Total number	
Male	
Female	
Age range	
Age median	
Age SD	

Source of patient sample (cases)

Source of patient sample (control)

Criteria for clinical diagnosis:

Country of study

Sex matched?   Yes/ No

Age matched?   Yes/No

Other matching:

EBV antibody	Assay type	% RA positive	% Cntrl positive	Odds ratio (raw)	Odds ratio (adj)
EBNA-1					
EBNA-2					
VCA					
EA					
Other					

NEWCASTLE - OTTAWA QUALITY ASSESSMENT SCALE
CASE CONTROL STUDIES (Modified)

Selection

1) Is the case definition adequate?
   - a) yes, use of American College of Rheumatology criteria or equivalent ✸
   - b) yes, eg record linkage or based on self reports
   - c) no description

2) Representativeness of the cases
   - a) consecutive or obviously representative series of cases ✸
   - b) potential for selection biases or not stated

3) Selection of Controls
   - a) community controls ✸
   - b) hospital controls
   - c) no description

4) Definition of Controls
   - a) no history of disease (endpoint) ✸
   - b) no description of source

Comparability

1) Comparability of cases and controls on the basis of the design or analysis
   - a) study controls for age ✸
   - b) study controls for any additional factor ✸

Exposure

1) Blinding of analysis
   - structured interview where blind to case/control status (max 2 stars)
     - i) conducting the analysis in a clinical laboratory (away from investigators) ✸
     - ii) mentioning explicit laboratory cut-offs for sero-positivity ✸

2) Same method of ascertainment for cases and controls.
   - a) yes✸
   - b) no

3) Missing data reported
   - a) yes ✸
   - b) no

NEWCASTLE - OTTAWA QUALITY ASSESSMENT SCALE
